# Shared Genetics Between Age at Menopause, Early Menopause, POI and Other Traits

**DOI:** 10.3389/fgene.2021.676546

**Published:** 2021-09-29

**Authors:** Yvonne V. Louwers, Jenny A. Visser

**Affiliations:** ^1^ Division of Reproductive Endocrinology and Infertility, Department of Obstetrics and Gynecology, Erasmus MC, University Medical Center Rotterdam, Rotterdam, Netherlands; ^2^ Department of Internal Medicine, Erasmus MC, University Medical Center Rotterdam, Rotterdam, Netherlands

**Keywords:** menopause, premature ovarian insufficiency, primary ovarian insufficiency, postmenopause, genetics, genome-wide association study, genetic pleiotropy, mendelian randomization analysis

## Abstract

Reproductive ageing leading to menopause is characterized by depletion of follicles and its regulating mechanisms are only partly understood. Early age at menopause and premature ovarian insufficiency (POI) are associated with several other traits such as cardiovascular disease, dyslipidemia, osteoporosis and diabetes. In large cohorts of Northern European women hundreds of Single Nucleotide Polymorphisms (SNPs) have been identified to be associated with age at menopause. These SNPs are located in genes enriched for immune and mitochondrial function as well as DNA repair and maintenance processes. Genetic predisposition to earlier menopause might also increase the risk of other associated traits. Increased risk for cardiovascular disease in women has been associated with age at menopause lowering SNPs. Pleiotropy between early age at menopause and increased mortality from coronary artery disease has been observed, implicating that genetic variants affecting age at menopause also affect the risk for coronary deaths. This review will discuss the shared genetics of age at menopause with other traits. Mendelian Randomization studies implicate causal genetic association between age at menopause and age at menarche, breast cancer, ovarian cancer, BMD and type 2 diabetes. Although the shared biological pathways remain to be determined, mechanisms that regulate duration of estrogen exposure remain an important focus.

## Introduction

Menopause is defined as the cessation of reproductive function and age at menopause is determined retrospectively as the absence of menses for 12 months (te Velde and Pearson, 2002). The mean age of natural menopause (ANM) in women of European descent is 51 years, showing a normal distribution ranging from 40 to 60 years (te Velde and Pearson, 2002). An early menopause (EM) before the age of 45 years occurs in ∼5% of women in the general population. Menopause before the age of 40 years is present in 1% of women and is considered a pathological condition referred to as premature ovarian insufficiency (POI) (Coulam et al., 1986; Webber et al., 2016). Importantly, already prior to menopause fecundity begins to decline (te Velde and Pearson, 2002). The latter has gained more interest given that women in the Western world decide to have their first child later in life, and thereby increasing the risk of age-related involuntary infertility (Broekmans et al., 2009).

Menopause results from exhaustion of the follicle pool. After the formation of the primordial follicle pool *in utero*, the ovary contains on average one million follicles at birth. Primordial follicles are continuously activated and recruited into the growing follicle pool of which, after puberty, only one is destined to reach the preovulatory stage. When the pool of primordial follicles is exhausted to 100–1,000 follicles, this number is too low to support reproductive cycles. In addition to the decline in follicle number, oocyte quality declines with increasing age (Hall, 2015). This reproductive aging also leads to changes in the hypothalamic-pituitary axis. The reduction in number of growing follicles results in decreased levels of inhibin B, estradiol and progesterone, and thereby loss of the negative feedback on GnRH pulse frequency and secretion and subsequently in significantly increased gonadotropin levels. In addition to ovarian aging, hypothalamic and pituitary aging have also been proposed to contribute to reproductive aging (Hall, 2015).

POI is often considered the result of premature ovarian aging, as a consequence of a reduced primordial follicle pool at birth, accelerated depletion of the follicle pool, or increased atresia of follicles. However, POI can also develop despite follicles being present due to insensitivity to gonadotropins. Therefore, POI can present as primary amenorrhea or as secondary amenorrhea, with the latter more closely resembling the process of natural menopause (Visser et al., 2012).

**FIGURE 1:**
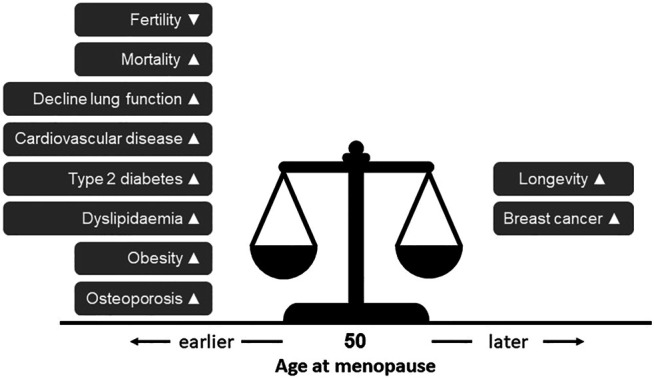
The impact of age at menopause on women’s health. Data of numerous conventional epidemiological studies indicate that the timing of age of menopause increases (▲) or decreases (▼) the risk for various diseases.

EM and POI not only lead to infertility but also increase the risk for osteoporosis, type 2 diabetes and cardiovascular disease, among others (Forman et al., 2013), as shown by numerous epidemiological studies (Figure 1). The lower estrogen levels are considered an important contributing factor to this increased risk. In contrast, an earlier age at menopause reduces the risk of breast cancer (Figure 1), possibly due to a reduced period of estrogen exposure (Forman et al., 2013).

## Genetics of Menopause, Early Menopause and POI

Age of natural menopause is determined by genetic and environmental factors. Particularly smoking has been demonstrated to be an important factor negatively impacting ANM (Forman et al., 2013). However, the genetic component in ANM is relatively strong, as twin studies have estimated that the heritability of ANM varies between 44 and 85% (te Velde and Pearson, 2002). Nevertheless, previous linkage studies have only been moderately successful in identifying genes influencing ANM, which can be explained by the fact that this technique is more suited to detect variants with large effect sizes. Candidate gene association studies have been more successful, although conflicting results have been reported and replication in independent cohorts is mostly lacking. For candidate gene studies, an a priori assumption is made that the selected gene is involved in the trait of analysis. Thus for ANM, genes involved in sex steroid biosynthesis and signaling, gonadotropin biosynthesis and signaling, and folliculogenesis have been studied mostly. Indeed, SNPs in FSHB and ESR1 showed consistent associations with ANM [reviewed in (Huhtaniemi et al., 2018; Jiao et al., 2018)]. A major limitation of candidate gene association studies is that it does not identify novel genes or pathways. This limitation is overcome with genome wide association studies (GWASs), which have an hypothesis-free approach (Hirschhorn and Daly, 2005). Indeed, GWAS of ANM have identified previously unpredicted genes and pathways, such as genes implicated in DNA damage response (DDR), immune function and mitochondrial biogenesis (Stolk et al., 2012; Day et al., 2015). Our GWAS meta-analysis of age at menopause, in nearly 70,000 women with European ancestry, identified 54 independent signals located in 44 genomic regions, with minor allele frequency (MAF) ranging between 7 and 49% (Day et al., 2015). Effect sizes ranged from 0.07 to 0.41 years per allele, with one SNP having an effect of 0.88 years. There was no clear pattern whether less frequent variants had a larger effect size, and the ANM lowering effects were observed for both minor and major alleles (Day et al., 2015). These 54 genome-wide significant SNPs explain 6% of the variance in ANM. Pathway analysis again highlighted a role for DDR pathways in age of menopause as nearly two-third of the ANM SNPs are involved in these pathways. Furthermore, five of the identified loci contain genes involved hypothalamic-pituitary function, including *FSHB*, suggesting also a neuro-endocrine component of ovarian aging (Day et al., 2015). In our latest GWAS in over 200,000 women of European ancestry, the number of ANM-genetic loci increased to 290 and strengthened the involvement of DDR pathways in the regulation of age of menopause (Ruth et al., 2021). These GWAS-ANM loci identified in women of European descent were also identified in cohorts of diverse ethnicities suggesting shared genetics in reproductive aging among ethnic groups (Chen et al., 2014; Fernández-Rhodes et al., 2018; Horikoshi et al., 2018; Zhang et al., 2021). However, assessment of the 290 ANM loci in a cohort of approximately 78,000 women of East Asian ancestry also highlighted the presence of heterogeneity in effect sizes and allele frequencies (Ruth et al., 2021).

To date four GWASs (Kang et al., 2008; Knauff et al., 2009; Pyun et al., 2012; Qin et al., 2012; Park et al., 2020) and two genome-wide linkage analyses (Oldenburg et al., 2008; Caburet et al., 2012) have been performed to identify POI-associated loci. However, cohorts of POI are relatively small and a GWAS may therefore lack sufficient statistical power. Indeed, only one genome-wide significant locus was identified in these studies (APBA3) (Park et al., 2020), and a few suggestive significant associations were observed (ADAMTS19; LAMC1; a region at 8q22.3) (Knauff et al., 2009; Pyun et al., 2012; Qin et al., 2012). Untangling the genetics of POI is further complicated by the fact that POI has syndromic and non-syndromic presentations, making POI a heterogeneous disease. However, recent technological advancement in Next Generation Sequencing (NGS), in particular Whole Exome Sequencing (WES), has led to the identification of novel causative genes in POI. Combined with previous candidate approaches, mutations in >60 genes have currently been identified (Huhtaniemi et al., 2018; Jiao et al., 2018). Interestingly, the increasing list of genes identified by WES shows enrichment of genes involved in DDR, homologous recombination, and meiosis (reviewed in (Huhtaniemi et al., 2018; Jiao et al., 2018)). This suggests that similar biological pathways may underlie POI and menopause. This hypothesis is supported by the finding that GWAS-ANM loci are enriched in genes linked to monogenic POI (Day et al., 2015). Other studies also reported shared genetics between EM and POI with ANM (He et al., 2010; Murray et al., 2011; Perry et al., 2013). Furthermore, in our study the combined effect of ANM lowering SNPs was estimated to explain 30% of the variance in EM (Perry et al., 2013). Thus, ANM, EM and POI may have an overlapping polygenic etiology, with women with POI carrying more ANM lowering variants and representing the extreme of the trait (Day et al., 2015; Ruth et al., 2021). The enrichment of DDR genes in ANM, EM, and POI furthermore suggests that reproductive aging may be part of systemic aging, as accumulation of DNA damage has been shown to be a major driver of aging (López-Otín et al., 2013). Thus, factors underlying genetic predisposition to earlier menopause might also be involved with other traits.

## Shared Genetics Between Age at Menopause and Other Traits

Improved knowledge of a shared genetic background between complex traits and diseases can highlight specific biological mechanisms underlying those traits and can identify causal relationships (Bulik-Sullivan et al., 2015a). Over the last 2 decades an enormous effort has been made to further identify common genetic variants in relation to complex traits, such as ANM. Large studies have been conducted analyzing hundreds of thousands of individuals which has led to the successful identification of tens of thousands of genetic variants associated with complex traits (Visscher et al., 2017). As a consequence of the identification of these genetic variants, studies are more and more focusing on further elucidating the complex interplay between all these associated genetic variants with different traits.

Several methods are available to further explore whether different traits have a shared underlying genetic mechanism. One of these, is to estimate the genetic correlation between traits, also interpreted as “shared heritability” (i.e., the correlation between the underlying genetic variance of the traits). Such genetic correlation is estimated from summary statistics using different methods. One of those, is based on regressing the association test statistics against a linkage disequilibrium (LD) score or LD-score regression (Bulik-Sullivan et al., 2015b). LD Score regression assumes that the GWAS effect size estimate for a given SNP reflects the effects of all SNPs in LD with that SNP (Bulik-Sullivan et al., 2015a). While the method was initially developed to differentiate “polygenicity” from “stratification,” the use of the regression can be extended to the estimation of heritability and genetic correlation (when evaluating multiple traits).

**FIGURE 2:**
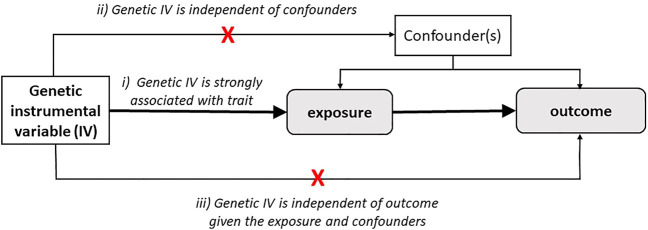
Schematic representation of Mendelian Randomization strategy. Mendelian randomization estimates the causal relationship of the trait (exposure) on other traits (outcome) through genetic variants as instrument variables (IVs). MR studies rely on three main assumptions: i) IVs are strongly associated with the exposure, ii) IVs are not associated with confounders, and iii) IVs affect the outcome solely through the exposure.

With the advent of numerous well-powered GWAS, genetic risk score (GRS) or polygenic risk scores (PRS) have leveraged their use as instrumental variables (IV) in the context of Mendelian randomization (MR). MR is an approach allowing to scrutinize evidence for causal relationships among associations between two traits in an observational setting. Figure 2 illustrates schematically the use of significantly associated SNPs as instrumental variables (IV), in the context of an MR setting and its underlying assumptions (discussed below) (Pasaniuc and Price, 2017; Davey Smith and Hemani, 2014; Davey Smith and Ebrahim, 2003). Mendel stated that inheritance of one trait is independent of the inheritance of other traits, in other words, there is a natural randomization of known and unknown confounders. MR is an effective method when instruments are robust, i.e., hold robust associations between SNPs and traits/diseases arising from well-powered settings (Bulik-Sullivan et al., 2015a). MR can be a reliable test to infer evidence for causal associations provided that several IV assumptions are met. These assumptions are shown in *italic* in Figure 2: 1) IVs should be strongly associated with the trait, 2) IVs share no common cause with the outcome, and 3) IVs affect the outcome solely through the exposure (absence of horizontal pleiotropy). Horizontal pleiotropy occurs when a certain SNP influences two traits independently, which constitutes a critical violation of the MR assumption and as such a threat for the validity of MR studies. The MR-Egger method has been proposed as an adequate method to evaluate whether there is evidence of bias-generating pleiotropy invalidating the genetic instrumental variables. Thus, it is important that sensitivity analyses and adequate methodology is used to verify compliance of the MR assumptions (Bulik-Sullivan et al., 2015a).

Below we will review examples of studies applying these methods to infer causal relationships between ANM and other health-related traits. The results of the MR studies are summarized in Table 1, in which we also indicated whether sensitivity tests were applied and reported.

**TABLE 1:** Overview of mendelian randomization studies for age at natural menopause.

Cohorts	Genetic variants with associated exposure	Outcome	MR method	Causal effect estimate	*p*	Ref	Sensitivity tests
Summary data from GIANT consortium	54 SNPs - ANM	BMI	Bidirectional-MR: weighted genetic risk score	Not reported	0.668	Day et al. (2015)	Not reported
Summary data from Reprogen consortium	32 SNPs - BMI	ANM	Bidirectional-MR: weighted genetic risk score	Not reported	0.683		
	54 SNPs - ANM	Age at menarche	Bidirectional-MR: weighted genetic risk score	Not reported	0.571		
	123 SNPs - age at menarche	ANM	Bidirectional-MR: weighted genetic risk score	Not reported	0.0005		
	56 SNPs - ANM	Breast cancer risk	Unconditional logistic regression	OR = 1.064; 95%CI 1.050–1.081	2.78 × 10^−14^		
Summary data from Reprogen consortium	210 SNPs - ANM	BMI	Two-sample MR: IVW method	β = −0.003; 95%CI -0.008–0.003	3.5 × 10^−1^	Ruth et al. (2021)	Yes
	227 SNPs - ANM	CAD	Two-sample MR: IVW method	OR = 1.002; 95%CI 0.992–1.013	6.5 × 10^−1^		
	229 SNPs - ANM	Breast cancer risk	Two-sample MR: IVW method	OR = 1.035; 95%CI 1.027–1.042	3.7 × 10^−17^		
	256 SNPs - ANM ER negative set	Breast cancer risk	Two-sample MR: IVW method	OR = 1.015; 95%CI 1.002–1.029	2.1 × 10^−2^		
	227 SNPs - ANM ER positive set	Breast cancer risk	Two-sample MR: IVW method	OR = 1.041; 95%CI 1.032–1.05	2.7 × 10^−16^		
	223 SNPs - ANM	Ovarian cancer risk	Two-sample MR: IVW method	OR = 1.028; 95%CI 1.013–1.043)	2.9 × 10^−4^		
	194 SNPs - ANM	Type 2 diabetes	Two-sample MR: IVW method	OR = 0.981; 95%CI 0.97–0.992	1.1 × 10^−3^		
	117 SNPs - ANM	Fasting glucose	Two-sample MR: IVW method	β = 0; 95%CI -0.006–0.005	8.9 × 10^−1^		
	127 SNPs - ANM	Fasting insulin	Two-sample MR: IVW method	β = −0.004; 95%CI -0.01–0.002	1.6 × 10^−1^		
	212 SNPs - ANM	Fracture risk	Two-sample MR: IVW method	OR = 0.983; 95%CI 0.974–0.992	4.7 × 10^−4^		
	243 SNPs - ANM	BMD (45–60 years)	Two-sample MR: IVW method	OR = 0.033; 95%CI 0.021–0.045	6.9 × 10^−8^		
Summary data from Reprogen consortium	298 SNPs - age at menarche	ANM	Two-sample MR: IVW method	β = 0.13; 95%CI 0.086–0.174	2.0 × 10^−8^		
Summary data from UK Biobank	110 SNPs - BMI	ANM	Two-sample MR: IVW method	β = −0.05; se = 0.01	0.027	Ding et al. (2020)	Yes
	111 SNPs - age at menarche	ANM	Two-sample MR: IVW method	β = 0.34; se = 0.16	0.035		
Summary data from Reprogen consortium	61 SNPs - age at menarche	ANM	Two-sample MR: IVW method	β = 0.23; se = 0.07	0.001		
Summary data from Social Science Genetic Association Consortium	16 SNPs - education	ANM	Two-sample MR: IVW method	β = 1.19; se = 0.41	0.004		
Summary data from UK Biobank	2 SNPs - smoking	ANM	Two-sample MR: IVW method	β = 0.26; se = 1.46	>0.05		
3 cohorts part of CHARGE consortium	54 SNPs - ANM	CAD	Not clearly reported	Not reported	0.52	Sarnowski et al. (2018)	Not reported
Summary data from Reprogen consortium	54 SNPs - ANM	Rheumatoid arthritis	Two-sample MR: IVW method	OR = 1.05; 95%CI 0.98–1.11	0.15	Zhu et al. (2021)	Yes
WHI data	2 SNPs - ANM (rs11668344; rs16991615	Accelaration epigenetic aging	IVW method	β = 0.506 (rs11668344)	0.031	Levine et al. (2016)	Not reported
				β = 0.151 (rs16991615)	0.763		
Summary data from Reprogen consortium	34 SNPs - ANM	Breast cancer survival	Two-sample MR: IVW method	HR = 1.01; 95%CI 0.98–1.05	0.49	Escala-Garcia et al. (2020)	Yes
	Premenopausal set	Breast cancer survival	Two-sample MR: IVW method	HR = 1.03; 95%CI 0.698–1.08	0.21		
	Postmenopausal set	Breast cancer survival	Multivariable model	HR = 1.01; 95%CI 0.93–1.08	0.9		
Summary data from Reprogen consortium; 3 consortia: GECCO. CCFR. CORECT (12,944 female cases. 10,741 female controls)	51 SNPs - ANM as GRS (risk increasing alleles)	Colorectal cancer	Two-sample MR: IVW method	OR = 0.99; 95%CI 0.83–1.17	—	Neumeyer et al. (2018)	Yes
Summary data from Reprogen consortium	54 SNPs - ANM	Fracture risk	Two-sample MR: IVW method	OR = 1.10; 95%CI 1.00–1.21	0.05	Trajanoska et al. (2018)	Yes
		Femoral Neck BMD	Two-sample MR: IVW method	EE = −0.063; 95%CI -0.080–0.047	0.038		
		Lumbar Spine BMD	Two-sample MR: IVW method	EE = −0.018; 95%CI -0.033–0.004	0.01		
UK Biobank	63 SNPs - ANM (39 strong IV for EM; 40 strong IV for late ANM)	Lung function FEV1/FVC(%) (EM)	Two-sample MR: IVW method	β = 0.29; 95%CI 0.22–0.36	1.48 × 10^-16^	van der Plaat et al. (2019)	Yes
		Lung function FEV1/FVC < LLN (EM)	Two-sample MR: IVW method	OR = 0.85; 95%CI 0.82–0.89	5.88 × 10^−14^		
		Lung function FEV1/FVC(%) (late ANM)	Two-sample MR: IVW method	β = −0.18; 95%CI -0.26–0.1	1.09 × 10^−5^		
		Lung function FEV1/FVC < LLN (late ANM)	Two-sample MR: IVW method	OR = 1.06; 95%CI 1.01–1.11	0.018		
Summary data from Reprogen consortium	35 SNPs -ANM	Invasive epithelial ovarian cancer	Two-sample MR: IVW method	OR = 1.03; 95%CI 1.00–1.06	0.07	Yarmolinsky et al. (2019)	Yes
				Ovarian cancer histotypes: endometriod	Two-sample MR: IVW method			OR = 1.19; 95%CI 1.05–1.36	0.008

### Metabolic Traits

#### Obesity and Waist Hip Circumference

Menopause is accompanied by an elevated prevalence of obesity (Davis et al., 2012). Menopause does not necessarily lead to weight gain, but it does cause an increase in total body fat and a redistribution of body fat, resulting in increased visceral adiposity (Davis et al., 2012; Tchernof and Després, 2013; Davis et al., 2015). Development of obesity after menopause seems the result of several factors, namely reduced physical activity and reduced energy expenditure, whether or not accompanied by depression, in combination with muscle atrophy and a lower basal metabolic rate (Davis et al., 2012). Whether obesity influences the timing of menopause is less well established. A large pooled analysis of prospective studies found that women with underweight (BMI <18.5 kg/m^2^) had a higher risk of EM (RR 2.15, 95% CI 1.50–3.06), while women with overweight (BMI 25–29.9 kg/m^2^) or obesity (BMI ≥30 kg/m^2^) more often had a later menopause (RR 1.52, 1.31–1.77 and RR 1.54, 1.18–2.01, respectively) compared to women with a normal BMI (Zhu et al., 2018). This suggest some sort of protective effect of obesity for EM. However, moderate heterogeneity between studies, self-reported BMI, and potential confounders including smoking, could influence these results. In contrast, genetic studies using cross-trait LD score regression have shown a significant negative genetic correlation between ANM and obesity and additional related anthropometric traits such as BMI and waist circumference in women only. ANM lowering SNPs were associated with higher BMI levels (Day et al., 2015; Sarnowski et al., 2018). Furthermore, a GRS for adult BMI increasing variants with BMI profiles from early to late adulthood showed that this association was strongest in women with EM (Song et al., 2018). However, (bidirectional) MR analysis did not identify evidence for a causal relationship between ANM and BMI (Day et al., 2015; Ruth et al., 2021; Sarnowski et al., 2018), while a study of Ding et al. suggests that there is genetic support for a causal association between higher BMI and earlier ANM (Ding et al., 2020). These contrasting results are likely due to differences in SNP selection and differences in sample size. Whether appropriate sensitivity analyses were performed, was not reported for all studies (Table 1). These data support the existence of shared biological mechanisms between ANM and BMI, however ANM lowering variants may not be causal for higher BMI. Importantly, the observed association between ANM and BMI by the MR study of Ding et al. (2020) shows an opposite direction than observed in the epidemiological study by Zhu et al. (2018). Assessment of BMI before or after menopause may account for this difference. This raises an interesting question whether BMI could be both a cause and consequence of ANM, which should be taken into account in the study design.

#### Lipid Regulation

Menopause transition results in lipid profile changes, with a 10–15% higher LDL-cholesterol and triglyceride levels and slightly lower HDL cholesterol levels (Choi et al., 2015). In agreement, POI is associated with similar changes in lipid profile (Knauff et al., 2008; Gulhan et al., 2012). Hence, a shared genetic background for reproductive ageing and lipid regulation has been proposed. Indeed, we observed overlapping signals between ANM SNPs and proxies (r^2^ > 0.5) and SNPs influencing lipid levels, including genetic variants in STARD3, and the cross-trait LD score regression was nominally significant for HDL levels (Teslovich et al., 2010; Day et al., 2015). Although the same gene region is associated with ANM and lipid levels, it remains to be determined whether the same variant drives this association. Colocalization methods (Burgess et al., 2018) may need to be applied to confirm whether there is indeed shared genetic etiology between ANM and lipid levels. Whether loci for lipid levels are associated with ANM has not been addressed yet. However, when inferring causality, potential pleiotropy between ANM and lipid levels, as observed in our initial study, should be taken into account. This is underlined by the involvement of steroidogenic acute regulatory protein (STAR) genes in ANM (STAR and STARD3) (Day et al., 2015; Perry et al., 2015). The proteins encoded by these genes function as cholesterol-binding proteins and play a role in translocation of cholesterol to the inner mitochondrial membrane. Also, by facilitating the conversion of cholesterol into pregnenolone, they are key players in the acute regulation of steroid hormone synthesis (Reitz et al., 2008). Our recent MR analysis, however, did not support causality between ANM and lipid levels (Ruth et al., 2021).

#### Cardiovascular Disease

It had been well established that menopause transition is related to an increase in cardiovascular risk and a reduced quality of life, both requiring preventive care (Maas et al., 2021). The reduction in estrogen levels are considered to play a critical role in this as estrogens regulate vascular reactivity, blood pressure (BP), endothelial function and cardiac remodeling (Turgeon et al., 2006; Miller and Duckles, 2008; Menazza and Murphy, 2016; Maas et al., 2021). Women with POI have a shorter life expectancy than women with a late menopause due to cardiovascular disease and osteoporosis (Ossewaarde et al., 2005; Atsma et al., 2006; Muka et al., 2016). Indeed, a meta-analysis showed an increased risk of CVD not only for women with EM and POI, but for all women with an ANM before the age of 50 (Zhu et al., 2019). Each earlier year of menopause was associated with a 3% increased risk of cardiovascular disease (Zhu et al., 2019).

Genetic data of more than 22,000 men and women derived from three population-based cohorts were analyzed and related to development of their first cardiovascular event (Sarnowski et al., 2018). The authors composed a GRS consisting of genetic variants associated with EM and POI in large GWASs (Day et al., 2015). Sex-stratified analyses showed that this GRS increased the risk of coronary heart disease deaths in women while no effect was observed in men (Sarnowski et al., 2018). In women, a 1-unit decrease in genetically predicted ANM increased the hazard of coronary heart disease death by 12% and increased the risk of the combined endpoint (including myocardial infarction, stroke, congestive heart failure, or death from coronary heart disease) by 10% (Sarnowski et al., 2018). Even after adjusting for menopause timing, the GRS remained associated with time to the first cardiovascular event. This suggests genetic pleiotropy between ANM and coronary deaths. In other words, some SNPs affect ANM as well as coronary deaths (Sarnowski et al., 2018). However, similar to BMI, causal inference by MR analysis could not be detected (Sarnowski et al., 2018; Ruth et al., 2021).

#### Diabetes

EM and POI are an independent riskfactor for type 2 diabetes (Anagnostis et al., 2019). Also, patients with type 1 diabetes have menopause at an earlier age than controls (Dorman et al., 2001; Kjaer et al., 1992). However, two large cohort studies found contrary results and were unable to link type 1 diabetes to EM (Kim et al., 2016; Yarde et al., 2015). It is likely that in the more recent type 1 diabetes cohort studies better glycemic control was achieved than in the previous cohorts, explaining the relatively low occurrence of substantial microvascular disease (Thong et al., 2020). Follicle recruitment and growth could be negatively affected by chronic hyperglycaemia, thereby leading to an impaired ovarian function in type 1 diabetes. Advanced glycation end-products, which are enhanced upon hyperglycaemia, have been suggested to contribute to ovarian ageing (Thong et al., 2020; Codner et al., 2012). In our latest GWAS study we did observe genetic pleiotropy between the GWAS-ANM SNPs and proxies (r^2^ > 0.5) and GWAS-identified loci for fasting glucose (Day et al., 2015). Amongst the identified overlapping GWAS loci was the *GCKR* gene, suggesting that an altered glucokinase regulation and glucose sensing might be a shared genetic etiology for reproductive ageing, type 2 diabetes, cardiovascular disease and dyslipidaemia (Dupuis et al., 2010; Day et al., 2015; Perry et al., 2015). However, as discussed for lipid regulation, it remains to be determined whether the same variant drives this association. Particularly, since cross-trait LD score regression did not reveal a genetic correlation (Day et al., 2015), suggesting that the observed associations with ANM and fasting glucose are independent. Likewise, causal inference between ANM and fasting glucose or fasting insulin was not identified in our latest GWAS study, while a significant causal association with type 2 diabetes was observed (Ruth et al., 2021).

### Breast Cancer and Ovarian Cancer

There is very convincing epidemiological evidence of an inverse relationship between ANM and breast cancer risk. The development of breast cancer is associated with later ANM, while breast cancer incidence decreases substantially in women with EM (Monninkhof et al., 1999; Collaborative Group on Ho, 2012). In line with this epidemiological association, our genetic analysis showed that carrying more ANM increasing SNPs increased the risk for breast cancer (Day et al., 2015). Furthermore, Mendelian randomization analyses supports the existence of a causal relationship between a later age at menopause and breast cancer risk. For each 1-year increase in ANM, a 5–6% increase in breast cancer risk was observed (Day et al., 2015; Ruth et al., 2021). Given that DDR genes are enriched in the association with ANM, this causal relationship between ANM and breast cancer may seem counterintuitive as entering menopause at a later age may reflect having more efficient DNA repair mechanisms (Laven et al., 2016). We therefore hypothesized that the increased susceptibility to breast cancer is not directly due to DNA repair mechanisms but rather results from longer exposure to sex steroids, established by a more efficient DDR pathway (Day et al., 2015; Laven et al., 2016). This is consistent with our observation that significantly larger effect estimates were found for ANM GRS in estrogen receptor (ER)-positive versus ER-negative breast cancer cases (Day et al., 2015). Furthermore, MR analysis stratified for ER-positive and ER-negative breast, only resulted in a significant causal inference for ER-positive cases (Ruth et al., 2021). Thus the causal genetic association between ANM and breast cancer risk appears complex, involving at least interactions between DDR pathways and estrogen signaling.

In addition to breast cancer risk, also breast cancer survival in relation to ANM has been studied. Earlier ANM has been associated with poorer breast cancer survival, where ANM did not (Orgéas et al., 2008). MR approach testing for a genetic association between breast cancer survival and ANM found no evidence for a causal relation (Table 1) (Escala-Garcia et al., 2020).

A later age at menopause, and thereby a longer period of exposure to sex steroids, has also been suggested to be a risk factor for ovarian cancer (Franceschi et al., 1991), although the number of epidemiological studies analyzing the association between ANM and ovarian cancer are scarce and show inconsistent results (Schildkraut et al., 2001). However, the identification of DDR genes in determining ANM, including BRCA1 and BRCA2 which are associated with both breast and ovarian cancer (Antoniou et al., 2003), warrants further analysis. MR analysis based on 35 ANM SNPs showed a trend in causal inference with epithelial ovarian cancer risk (Yarmolinsky et al., 2019). Furthermore, stratification based on ovarian cancer subtypes identified a significant causal association between ANM and endometriod ovarian cancer (Table 1) (Yarmolinsky et al., 2019). Increasing the number of ANM SNPs as instrument variables, as shown in our recent MR analysis, resulted in a significant causal association between ANM and ovarian cancer, with a later age of menopause increasing the risk of ovarian cancer (Table 1) (Ruth et al., 2021). These findings suggests that a potential genetic overlap exists between ANM and ovarian cancer risk. As for breast cancer risk, an interaction between estrogen signaling and DDR pathways may be the underlying biological mechanism.

### Osteoporosis

Estrogen deficiency results in increased bone remodeling leading to osteoporosis. The underlying biological mechanisms are increased bone resorption due to increased osteoclast activity and reduced bone formation by osteoblasts, and as a result, bone resorption exceeds bone formation. The net loss of bone due to remodeling upon estrogen deficiency is estimated to be around 2–3% per year after menopause (Webber et al., 2016). Development of osteoporosis is an important concern for women with EM and POI. Epidemiological studies found a prevalence of 8–27% of osteoporosis in women with POI (Francucci et al., 2008; Sullivan et al., 2017; Xu et al., 2020). In our GWAS study, we did not report on shared genetics between ANM and osteoporosis or related traits (Day et al., 2015). However, in a cohort of Han Chinese postmenopausal women, out of 22 osteoporosis SNPs, selected from a bone mineral density (BMD) GWAS, it was observed that a SNP in MHC was significantly associated with ANM after correction for multiple testing (Zhao et al., 2011). However, the MHC region is associated with a multitude of diseases (de Bakker and Raychaudhuri, 2012), and combined with the candidate gene-like approach, it thus remains to be determined whether the association of MHC SNP with ANM persists in an unbiased analysis. Indeed, while cross-trait LD Score regression did showed a negative genetic correlation between ANM and fracture risk, upon correction for multiple testing this failed to reach significance (Trajanoska et al., 2018). This study by Trajanoska et al. (2018) also revealed that based on MR there is no evidence for a causal effect of ANM SNPs on fracture risk, while only a marginal effect was observed for BMD. However, increasing the number of ANM SNPs as instrument variables did indicate a causal relationship between ANM increasing SNPs and fracture risk and BMD (Ruth et al., 2021). Thus, a genetic link between earlier ANM and osteoporosis or BMD may be present. Interestingly, shared genetic variants between bone mineral density (BMD) and other reproductive traits including age at menarche and puberty timing, were found (Christou et al., 2020). It has been shown though that shared genetic factors by BMD and age at menarche were only identified in premenopausal women but not in postmenopausal women (Zhang et al., 2009). This GWAS result suggests that ANM may be a confounding factor in this shared genetic background possibly by affecting the duration of estrogen exposure. However, it should be noted that this study was performed in a relatively small cohort of <3,000 pre- and postmenopausal women. Replication in well-powered sample sizes is therefore needed to unravel the relationship between ANM, age at menarche, and bone-related phenotypes.

### Age at Menarche and Puberty Timing

Several epidemiological studies have linked onset of menarche as well as puberty timing to ANM. Nine studies in the UK, Scandinavia, Australia and Japan including over 50,000 women observed that having menarche before the age of 11 years increased the risk of EM and POI compared to having menarche at 13 years (Mishra et al., 2017). The association of early menarche and EM was also observed in American and Chinese women (Wang et al., 2018; Whitcomb et al., 2018).

In line with the above, while initial GWAS observed that ANM loci were different from those for age at menarche (He et al., 2009), subsequent GWAS analysis show that they partially overlap with genes implicated in disorders of puberty (Perry et al., 2014; Day et al., 2015; Perry et al., 2015). These SNPs are located in or near genes involved in the regulation of the hypothalamic–pituitary–gonadal axis (such as CHD7, FGFR1, SOX10, KISS1 and TAC3) (Day et al., 2015), suggesting that these reproductive milestones are at least partly regulated by shared biological mechanisms (Perry et al., 2015). Indeed, our cross-trait LD Score regression identified a genetic correlation between these two traits (Day et al., 2015), and a causal relationship was implied in recent MR analyses showing that a later age of menarche is associated with a later ANM (Ding et al., 2020; Ruth et al., 2021). Knowledge of this shared genetics of ANM and age at menarche may proof to be important when analyzing traits impacted by the duration of estrogen exposure, which applies to many of the postmenopausal diseases.

### Other Traits

In this paragraph we describe other traits for which the association with ANM has been studied in observational epidemiological studies and for which the causal relationship was further explored using the MR approach or GRS strategy.

The relationship between SNPs associated with ANM and polycystic ovary syndrome (PCOS), as well as reproductive markers such as gonadotrophin levels and ovarian volume, has been studied using a GRS strategy. One small longitudinal study suggested that PCOS is associated with entering menopause at a later age (Mulders et al., 2004). Throughout their entire life, women with PCOS encounter a larger number of follicles than women without PCOS at similar age (Mulders et al., 2004). This, together with a potential later ANM, raises the hypothesis that SNPs associated with ANM might also be associated with risk for PCOS. Indeed, analysis in a discovery and validation cohort showed that ANM increasing genetic variants were associated with risk for PCOS (Saxena et al., 2015). Furthermore, ANM lowering SNPs yield an opposing effect on ANM in women with PCOS, when corrected for BMI. This opposing effect could be partly explained by the association of ANM SNPs with higher luteinizing hormone (LH) levels (Saxena et al., 2015). It should be noted that in this study the GRS was calculated from a limited number of ANM variants, based on initial GWAS studies. It would be interesting to repeat this study using the SNPs from the latest GWAS study on ANM.

Observational studies suggest that lower forced vital capacity (FVC) and a higher risk of spirometric restriction is associated with an earlier age at menopause. A Mendelian randomization study suggests that ANM lowering SNPs were associated with a 15% lower risk of spirometric restriction (van der Plaat et al., 2019). Entering menopause at an earlier age seems to have a positive effect on airflow obstruction (van der Plaat et al., 2019). Further studies are needed to investigate the underlying mechanisms and the role of female sex hormones in lung function.

Development of colorectal cancer seems inversely associated with the use of menopausal hormone replacement therapy (Lin et al., 2012). This suggests a role of prolonged estrogen exposure in colorectal cancer development. However, a relationship between endogenous estrogen exposure and colorectal cancer is inconclusive. A large Mendelian randomization study did not find a causal relationship between GRS for ANM and colorectal cancer risk (Neumeyer et al., 2018).

Several large epidemiologic studies have observed an association with earlier ANM and increased risk of rheumatoid arthritis (Karlson et al., 2004; Bengtsson et al., 2017). Methodologically solid MR analysis by Zhu et al. did not support a causal relationship between ANM and the development of rheumatoid arthritis (Zhu et al., 2021).

A relationship between ANM and biological aging rate has been proposed. Data from four large observational studies found an association between ANM and methylation in blood as a marker of epigenetic aging (Levine et al., 2016). The authors also conducted a well-designed MR approach in which they selected the two most significant GWAS ANM SNPs as IVs. One of these SNPs also showed a significant association with epigenetic age acceleration (Levine et al., 2016). However, whether evidence for causal inference would be obtained using the complete GWAS summary statistics was unfortunately not addressed. MR analysis using our recent identified ANM SNPs did not infer causality between ANM and longevity (Ruth et al., 2021). Given that the DDR pathway has been implicated in longevity (Debrabant et al., 2014; Vermeij et al., 2014) this raises the question whether different DNA repair mechanisms are involved in reproductive aging and longevity.

## Conclusion

Conventional observation studies have identified several associations between POI, early menopause as well as menopause and other traits. However, confounding, reverse causation and other potential bias complicate translation of these associations into causal inference. Large GWAS studies have been performed in which hundreds of SNPs are found to be associated with the abovementioned traits. Advanced genetic approaches like MR are a promising way of identifying causality provided that all the required assumptions are met. It is therefore important that the statistical methods for IV analysis and several sensitivity tests are clearly reported.

**FIGURE 3:**
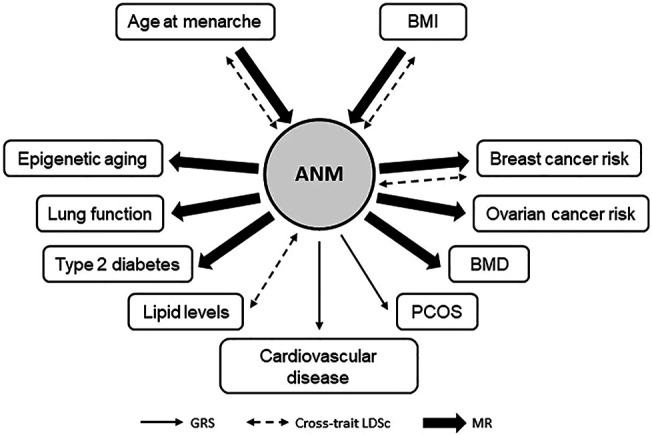
Mendelian randomization studies for age at natural menopause. Schematic summary of MR studies for age at natural menopause (ANM) with other health-related traits using ANM SNPs as instrument variables. Only traits with a significant result are depicted.

The studies discussed in this review (significant findings are summarized in Figure 3) suggest that shared genetics may exist between ANM and cardiovascular disease and dyslipidemia, although evidence for causal associations were not observed. For the well-known epidemiological association between ANM and osteoporosis, evidence for a causal genetic relationship was identified when the number of ANM SNPs as instrument variables was largely increased. The same was observed for type 2 diabetes. Based on studies published to date, genetic associations between ANM and age at menarche, breast cancer, and ovarian cancer risk appear causal and robust. Biological mechanisms that regulate duration of estrogen exposure therefore remain an important focus. The negative correlation between ANM and BMI, potentially causal, suggests that BMI is an important confounding factor that could impact the validity of MR studies. The majority of the genetic analyses for ANM discussed in this review have analyzed cross-trait associations in women only. Only two studies, analyzing the association of ANM with BMI and cardiovascular disease, used a sex-stratified approach, and observed associations in women only. While more studies are needed and causality remains to be determined, this may suggest that the biological pathways underlying menopause-related traits are women-specific.

It should be noted that the reported causal associations have been mostly identified in cohorts of European descent. However, the timing of menopause differs by geographical region, race/ethnicity and socioeconomic status (El Khoudary, 2020). Indeed, although shared genetics in ANM among different ethnic groups were reported, differences in allele frequencies and effect sizes were also observed. Furthermore, the risk for post-menopausal traits, such as breast cancer, CVD, and osteoporosis, differs between ethnicities (Cauley, 2011; Danforth Jr, 2013; Bays et al., 2021). Hence, it is suggested that valid causal estimates are obtained when cohorts have a similar racial/ethnic background (Burgess et al., 2015). Although receiving increased attention, more studies are needed to identify transethnic causal associations between ANM and other traits.

These studies also highlight that a critical analysis of the genetic variants and methods used is important. We advocate for multidisciplinary research teams, consisting of epidemiologists, geneticists, clinicians and basic scientists, to allow a proper design and interpretation of the results. Although in its infancy, it is expected that these studies will further elucidate the shared genetics of menopause, early menopause, and POI with other traits and thereby provide new insights in shared biological pathways.
